# Circβ-catenin promotes tumor growth and Warburg effect of gallbladder cancer by regulating STMN1 expression

**DOI:** 10.1038/s41420-021-00626-6

**Published:** 2021-09-06

**Authors:** Shouhua Wang, Tingting Su, Huanjun Tong, Di Zhou, Fei Ma, Jun Ding, Yuan Hao, Weibin Shi, Zhiwei Quan

**Affiliations:** 1 Department of General Surgery, Xinhua Hospital, Shanghai Jiao Tong University School of Medicine, Shanghai, 200092 China; 2 Shanghai Key Laboratory of Biliary Tract Disease Research, Shanghai, 200092 China; 3 Department of Oncology, Xinhua Hospital, Shanghai Jiao Tong University School of Medicine, Shanghai, 200092 China; 4 Department of Biliary and Pancreatic Surgery, Shanghai Shuguang Hospital Affiliated with Shanghai University of T.C.M., Shanghai, 201203 China

**Keywords:** Cancer therapy, RNAi

## Abstract

Gallbladder cancer (GBC) is the most malignant cancer of the biliary tract cancer and presents poor prognosis. CircRNAs have been identified as critical regulators of multiple stages in tumor progression. In the study, we first demonstrated that circular RNA circβ-catenin expression was upregulated in GBC tissues when compared to adjacent normal tissues and associated with advanced clinical stage and poor prognosis in GBC patients. Silencing of circβ-catenin obviously suppressed GBC cell proliferation and cell cycle progression in vitro, but circβ-catenin overexpression had the opposite effects. In vivo, silencing of circβ-catenin inhibited tumor growth. Furthermore, we also found that circβ-catenin promoted GBC cell lactate production, pyruvate production, ATP quantity, and extracellular acidification rate (ECAR), which suggested that circβ-catenin regulated Warburg effect in GBC. Mechanistic analysis further highlighted that circβ-catenin promoted Stathmin 1 (STMN1) expression through sponging miR-223 in GBC progression. In addition, knockdown of STMN1 inhibited cell growth and Warburg effect in GBC. In summary, our findings indicated that circβ-catenin/miR-223/STMN1 axis could regulate cell growth and Warburg effect in GBC. Targeting circβ-catenin might be a potential therapeutic strategy for GBC.

## Introduction

Gallbladder cancer (GBC) is the fifth most common cancer among gastrointestinal cancers and the most common cancer of the biliary tract worldwide [1]. Due to the absence of specific symptoms and effective treatment strategies at advanced stage for GBC patients, less than 50% of preoperatively known gallbladder cancer patients are candidates for curative resection [2]. The mean overall survival for GBC patients is only 6 months, with poor 5-year survival rates [3, 4]. The exact molecular alteration underlying gallbladder cancer pathogenesis remains largely unknown and the prognostic markers or therapeutic strategies are urgently needed.

Circular RNAs are a novel group of endogenous noncoding RNAs and are characterized by their covalently closed-loop structures without a 5′ cap or a 3′ Poly A tail [5, 6]. Currently, circRNAs garnered more attention and are found to be associated with tumorigenesis as well as their potential as diagnostic and prognostic biomarkers for some human cancers [7, 8]. CircRNAs are considerable functional potential to be involved in biogenesis, functions, and clinical significance [9]. For examples, circular RNA_LARP4 (circLARP4) is downregulated in gastric cancer and sponges to miR-424 by circRNA expression profile and bioinformatic analysis and inhibits biological behaviors of gastric cancer by affecting LATS1 expression [10]. CircTP63 is significantly upregulated in lung squamous cell carcinoma tissues and could competitively bind to miR-873-3p, which promotes lung squamous cell carcinoma progression by regulating FOXM1 expression [11]. In gallbladder cancer progression, researchers also showed that a circRNA generated from the oncogene ERBB2, named as circERBB2 promotes gallbladder cancer progression by regulating PA2G4-dependent rDNA transcription [12].

Circβ-catenin originated from β-catenin gene locus is predominantly localized in the cytoplasm and has been revealed to promote liver cancer cell growth through activation of the WNT pathway [13]. In our study, we first demonstrated that circβ-catenin was upregulated in gallbladder cancer and predicted a poor prognosis in GBC patients. Furthermore, functional investigations showed that circβ-catenin promoted cell proliferation, cell cycle progression, and cell glycolysis in vitro, and knockdown of circβ-catenin inhibited cell growth in vivo. Subsequent studies displayed that circβ-catenin sponged to miR-223 to regulate STMN1 expression in GBC cells, which affected tumor progression. Thus, these evidences highlight that circβ-catenin could exist as a novel molecular biomarker and therapeutic target for GBC.

## Results

### Circβ-catenin expression is upregulated in GBC tissues and cells

**Fig. 1:**
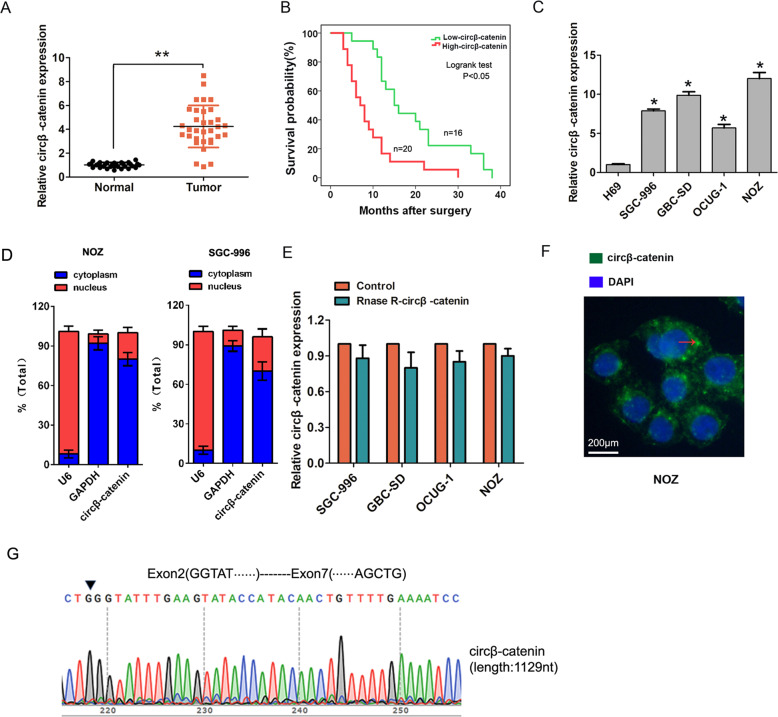
The circβ-catenin expression is upregulated in GBC tissues and cells. **A** The relative expression of circβ-catenin in gallbladder cancer tissues and adjacent normal tissues was detected by qRT-PCR analysis. **B** The correlation between the expression of circβ-catenin and over survival (OS) of GBC patients analyzed by the Kaplan–Meier method and log-rank test. **C** the relative expression of circβ-catenin in GBC cells (GBC-SD, SGC-996, NOZ, and OCUG-1) and H69 cell was determined by qRT-PCR analysis. **D** The expression of circβ-catenin was assessed by qRT-PCR in the nuclear and cytoplasm fractions in NOZ or SGC-996 cell lines. **E** RNase R was used to pretreat the RNAs and we found that the circular form of circβ-catenin was resistant to RNase R in four GBC cell lines compared with control group. **F** The subcellular localization of circβ-catenin detected by FISH assay. **G** RNA was isolated and RT-PCR with Sanger sequencing confirmed the circular form in NOZ cells and comes from exon regions. Data are shown as mean ± SD. *P < 0.05, **P < 0.01.

In order to investigate the clinical significance of circβ-catenin expression in GBC tissues and cells, we performed the qRT-PCR analysis. Our results found that circβ-catenin expression was markedly upregulated in human GBC tissues compared to adjacent normal tissues (Fig. 1A). Furthermore, we divided the GBC patients into two groups (higher circβ-catenin expression or lower circβ-catenin expression) according to the median expression of circβ-catenin in GBC tissues. Statistical analysis demonstrated that increased expression of circβ-catenin was significantly associated with advanced TNM stage (Table 1, *P* = 0.021), but no association with other clinicopathological characteristics (Table 1, *P* > 0.05). Moreover, Kaplan–Meier survival curve and log-rank test showed that GBC patients with higher expression of circβ-catenin have significantly shorter overall survival (OS) than those with the lower expression of circβ-catenin (Fig. 1B, log-rank test, *P* < 0.05). Multivariate Cox survival analysis also showed that higher circβ-catenin expression (hazard ratio [HR] = 2.577, 95% confidence interval [CI] 1.455–4.766, *P* < 0.01) and advanced TNM stage (hazard ratio [HR] = 2.233, 95% confidence interval [CI] 1.366–4.344, *P* < 0.01) were independent prognostic factors for poor survival of GBC patients (Table 2). In addition, circβ-catenin expression was also significantly higher in GBC cells (SGC-996, NOZ, GBC-SD, and OCUG-1) compared with a human intrahepatic biliary epithelial cell line H69 (Fig. 1C). Thus, these results indicated that circβ-catenin expression was upregulated in GBC and could act as a predictor of GBC prognosis.

**Table 1:** The association between circβ-catenin expression level and clinicopathological characteristics in 36 cases of GBC patients.

		circβ-catenin expression		
Clinicopathological characteristics	The number of patients (*n* = 36)	Lower (*n* = 16)	Higher (*n* = 20)	*P*-value
Age				0.877
≤60	23	10	13	
>60	13	6	7	
Sex				0.418
Male	11	6	5	
Female	25	10	15	
Tumor size				0.821
<5 cm	15	7	8	
≥5 cm	21	9	12	
Histological grade				0.709
Well and moderately	19	9	10	
Poorly and others	17	7	10	
Lymph node metastasis				0.051
Negative	16	10	6	
Positive	20	6	14	
TNM stage				0.021*
I–II	17	11	6	
III–IV	19	5	14	

*TNM* tumor-node-metastasis.

*P < 0.05.

**Table 2:** Multivariate Cox analysis of the overall survival (OS) in 36 GBC patients.

Factors	Multivariate Cox analysis		
	HR	95% CI	*P*-value
Age	0.863	0.655–1.125	0.902
Sex	1.002	0.343–1.366	0.589
Tumor size	1.012	0.655–1.789	0.688
Histological grade	1.208	0.556–2.088	0.445
Lymph node metastasis	1.488	0.936–3.066	0.166
TNM stage	2.233	1.366–4.344	0.004*
Higher circβ-catenin expression	2.577	1.455–4.766	0.001*

*HR* hazard ratio, *CI* confidence intervals.

*P < 0.05.

### Circβ-catenin promotes cell proliferation, cell cycle progression in vitro, and tumor growth in vivo

Next, to explore the potential role of circβ-catenin expression in GBC progression, circβ-catenin RNA expression in the nuclear and cytoplasmic fractions of NOZ and SGC-996 cells was detected by qRT-PCR. The results indicated that circβ-catenin was predominantly localized in the cytoplasm (Fig. 1D). RNase R was used to pretreat the RNAs and we found that the circular form of circβ-catenin was resistant to RNase R in four GBC cell lines compared with control group (Fig. 1E). Fluorescence in situ hybridization (FISH) assay also demonstrated that circβ-catenin predominately localized in the cytoplasm in NOZ cell (Fig. 1F). RNA was isolated and RT-PCR with Sanger sequencing confirmed the circular form in NOZ cells and comes from exon regions (Fig. 1G).

**Fig. 2:**
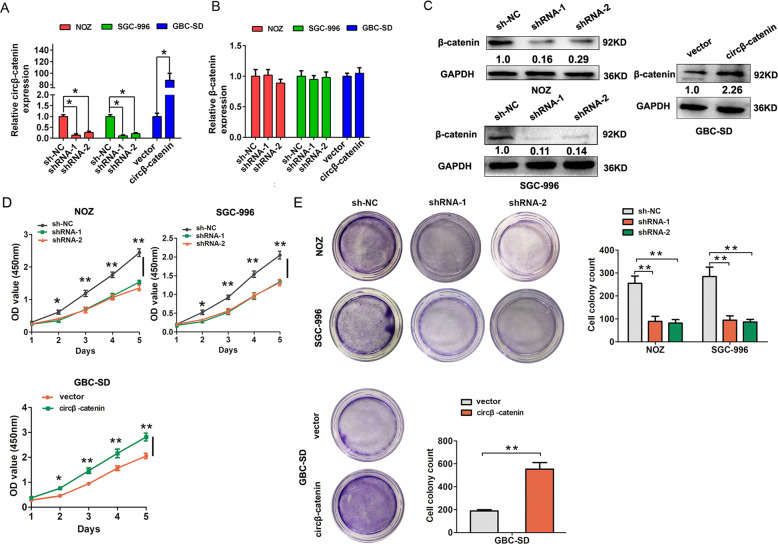
The circβ-catenin promotes cell proliferation in vitro. **A** The relative expression of circβ-catenin in NOZ and SGC-996 cells was detected by qRT-PCR analysis after cells were transfected with shRNA-NC, shRNA-1, or shRNA-2 or were transfected with pLCDH-vector or pLCDH-circβ-catenin in GBC-SD cells. **B** The relative expression of β-catenin in NOZ and SGC-996 cells was detected by qRT-PCR analysis after cells were transfected with shRNA-NC, shRNA-1, or shRNA-2, or were transfected with pLCDH-vector or pLCDH-circβ-catenin in GBC-SD cells. **C** The relative protein expression of β-catenin in NOZ and SGC-996 cells was detected by western blotting analysis after cells were transfected with shRNA-NC, shRNA-1, or shRNA-2, or were transfected with pLCDH-vector or pLCDH-circβ-catenin in GBC-SD cells. **D** The cell viability was detected by CCK8 assays in NOZ and SGC-996 cells after cells were transfected with shRNA-NC, shRNA-1, or shRNA-2, or were transfected with pLCDH-vector or pLCDH-circβ-catenin in GBC-SD cells. **E** The cell colonies number was detected in NOZ and SGC-996 cells after cells were transfected with shRNA-NC, shRNA-1, or shRNA-2, or were transfected with pLCDH-vector or pLCDH-circβ-catenin in GBC-SD cells. Data are shown as mean ± SD. *P < 0.05, **P < 0.01.

To analyze the potential functional role of circβ-catenin expression, we performed functional experiments by silencing circβ-catenin expression in NOZ and SGC-996 cells or overexpressed circβ-catenin in GBC-SD cells according to their expression in GBC cells (Fig. 2A). We also found that the two shRNAs or overexpressed circβ-catenin plasmid do not affect β-catenin mRNA expression but affect β-catenin protein expression (Fig. 2B, C). CCK8 assay demonstrated that circβ-catenin silencing significantly suppressed proliferation of NOZ and SGC-996 cells compared to the control group. However, circβ-catenin overexpression significantly enhanced cell proliferation in GBC-SD cells, compared to the control group (Fig. 2D). Colony formation assay also indicated that circβ-catenin knockdown decreased colony number of NOZ and SGC-996 cells, compared to the control group. However, circβ-catenin overexpression significantly increased cell colony number of GBC-SD cells (Fig. 2E).

**Fig. 3:**
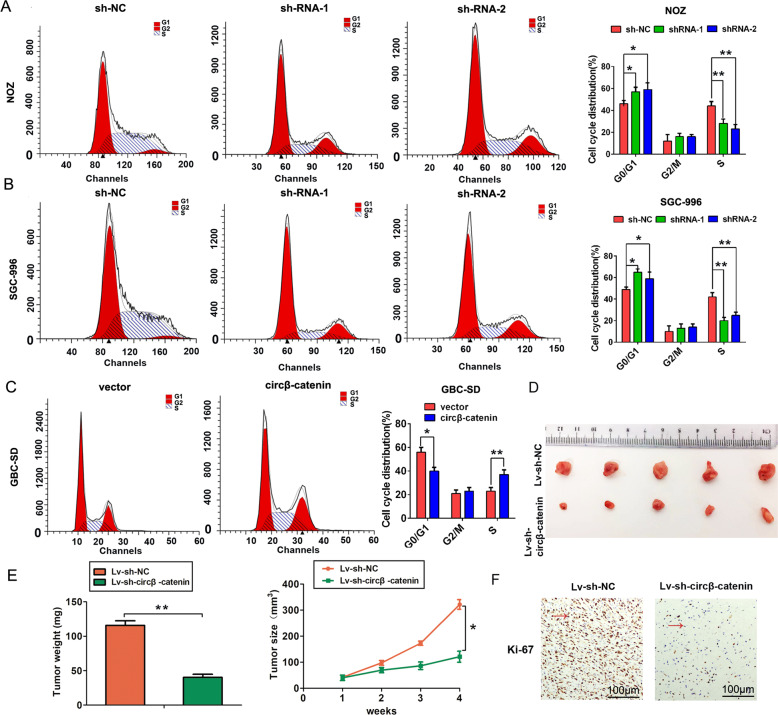
The circβ-catenin promotes cell cycle progression in vitro and tumor growth in vivo. **A**, **B** Flow cytometry analysis was shown as the percentage cell phase distribution including G0/G1, S, and G2/M phases after transfection of NOZ and SGC-996 cells with shRNA-NC, shRNA-1, or shRNA-2. **C** Flow cytometry analysis was shown as the percentage cell phase distribution including G0/G1, S, and G2/M phases after transfection of GBC-SD cells with pLCDH-vector or pLCDH-circβ-catenin. **D** Nude mice were subcutaneously injected with Lv-shRNA-NC cells or cells transfected with Lv-sh-circβ-catenin, after 4 weeks, tumors were dissected and imaged. **E** Tumor weight or volume was detected to monitor tumor growth in subcutaneous implantation mouse models transfected with Lv-shRNA-NC cells or cells transfected with Lv-sh-circβ-catenin. **F** Immunohistochemical staining of Ki-67 expression was shown in tumor tissues in Lv-shRNA-NC or Lv-sh-circβ-catenin group (original magnification, ×200). Data are shown as mean ± SD. *P < 0.05, **P < 0.01.

In addition, we detected the effect of circβ-catenin expression on the cell cycle by performing the flow cytometry analysis. Our results demonstrated that circβ-catenin knockdown dramatically decreased S phase cell number in NOZ and SGC-996 cells, compared to the control group. However, circβ-catenin overexpression significantly increased S phase cell number in GBC-SD cells, compared to the control group (Fig. 3A–C). In vivo, we established the xenograft mouse models by subcutaneously injecting an equal amount of NOZ cells. Results showed that circβ-catenin knockdown significantly suppressed tumor size and tumor volume compared to the control group (Fig. 3D, E). Immunohistochemical (IHC) analysis showed that circβ-catenin silencing also markedly decreased the Ki-67 expression compared to the control group (Fig. 3F). Thus, these results showed that circβ-catenin promoted cell proliferation, cell cycle progression in vitro, and tumor growth in vivo.

### Circβ-catenin promotes cell Warburg effect in GBC

**Fig. 4:**
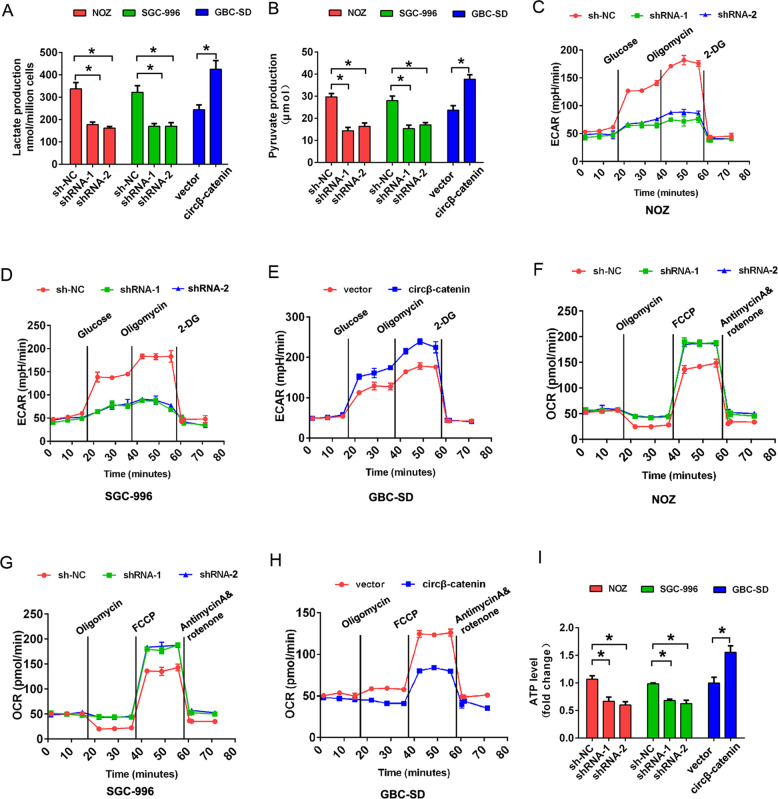
The circβ-catenin promotes cell glycolysis in GBC cells. **A**, **B** The lactate production and pyruvate production levels were analyzed after transfection of NOZ and SGC-996 cells with shRNA-NC, shRNA-1, or shRNA-2, or after transfection of GBC-SD cells with pLCDH-vector or pLCDH-circβ-catenin in GBC-SD cells. **C**–**E** The extracellular acidification rate (ECAR) analysis for mitochondrial respiratory capacity was conducted using a Seahorse XFp assay after transfection of NOZ and SGC-996 cells with shRNA-NC, shRNA-1, or shRNA-2, or transfection of GBC-SD cells with pLCDH-vector or pLCDH-circβ-catenin. **F**–**H** The Oxygen consumption rate (OCR) analysis for mitochondrial respiratory capacity was conducted using a Seahorse XFp assay after transfection of NOZ and SGC-996 cells with shRNA-NC, shRNA-1, or shRNA-2, or after transfection of GBC-SD cells with pLCDH-vector or pLCDH-circβ-catenin. **I** ATP quantity analysis revealed the ATP quantity after transfection of NOZ and SGC-996 cells with shRNA-NC, shRNA-1, or shRNA-2, or after transfection of GBC-SD cells with pLCDH-vector or pLCDH-circβ-catenin. Data are shown as mean ± SD. *P < 0.05.

Warburg effect is one of the hallmarks of cancers, characterized by the promotion of glycolysis and the inhibition of the oxidative phosphorylation with the presence of oxygen [14]. We investigated the effects of circβ-catenin on the Warburg effect of gallbladder cancer cells through measuring the lactate production, pyruvate production, extracellular acidification rate (ECAR), oxygen consumption rate (OCR), and ATP production after downregulation or upregulation of circβ-catenin. Our results found that circβ-catenin knockdown reduced lactate production and pyruvate production compared to the control group in NOZ and SGC-996 cells. However, circβ-catenin overexpression increased lactate production and pyruvate production in GBC-SD cells (Fig. 4A, B). Extracellular acidification rate (ECAR) analysis showed that circβ-catenin knockdown inhibited the extracellular acidification rate in NOZ and SGC-996 cells, but circβ-catenin overexpression promoted extracellular acidification rate in GBC-SD cells (Fig. 4C–E). Oxygen consumption rate (OCR) analysis for mitochondrial respiratory capacity showed that circβ-catenin knockdown increased the OCR compared to the control group in NOZ and SGC-996 cells, but circβ-catenin overexpression reduced OCR compared to the control group in GBC-SD cell (Fig. 4F–H). In addition, circβ-catenin knockdown reduced ATP quantity level compared to the control group in NOZ and SGC-996 cells, however, circβ-catenin overexpression promoted ATP quantity level in GBC-SD cell (Fig. 4I). These results suggested that circβ-catenin promoted Warburg effect in GBC cells.

### Circβ-catenin acts as a sponge for miR-223 in GBC

**Fig. 5:**
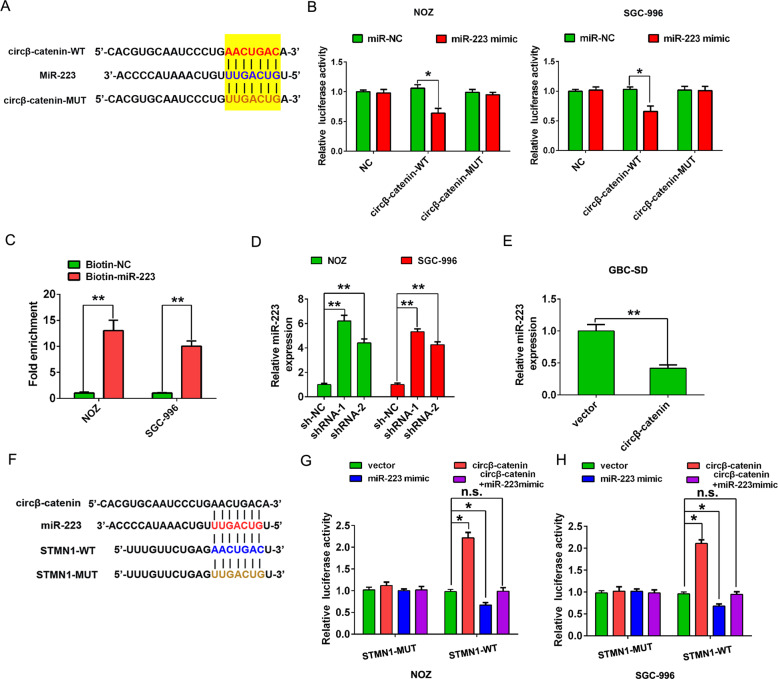
circβ-catenin targets miR-223/STMN1 in GBC cells. **A** MiR-223 showed complementary base pairing with circβ-catenin using the online software tools circinteractome (http://circinteractome.nia.nih.gov). **B** Luciferase reporter assay illustrated the correlation within circβ-catenin-WT and miR-223. **C** 3′-terminal-biotinylated-miR-223 probe that was verified to pull down circβ-catenin in NOZ and SGC-996 cells. **D**, **E** The miR-223 expression was detected by qRT-PCR after transfection of NOZ and SGC-996 cells with shRNA-NC, shRNA-1, or shRNA-2, or after transfection of GBC-SD cells with pLCDH-vector or pLCDH-circβ-catenin. **F** The WT and corresponding MUT of the STMN1 mRNA 3′ UTR were constructed targeting miR-223. **G**, **H** Luciferase reporter assays were performed in NOZ and SGC-996 cells transfected with pLCDH-vector, pLCDH-circβ-catenin, miR-223 mimic, or co-transfected with miR-223 mimic + pLCDH-circβ-catenin and STMN1-WT or STMN1-MUT reporter vector. Data are shown as mean ± SD. *P < 0.05. **P < 0.01. n.s., not significant.

Given that circRNAs have been reported to function as sponges for miRNAs [11]. Next, we performed a search for miRNAs that have complementary base pairing with circβ-catenin using the online software tools circinteractome (http://circinteractome.nia.nih.gov) and found that miR-223 could form complementary base pairing with circβ-catenin (Fig. 5A). To demonstrated the relationship between circβ-catenin and miR-223, we mutated the binding sites with miR-223 in circβ-catenin. The sequence of circβ-catenin containing wild-type (WT) or mutant type (MUT) binding sites with miR-223 was constructed (Fig. 5A). NOZ and SGC-996 cells were co-transfected with miR-NC or miR-223 mimic. As shown in Fig. 5B, the luciferase activity was dramatically reduced in circβ-catenin-WT group compared to the control group, while the luciferase activity remained unaffected in circβ-catenin-MUT in NOZ or SGC-996 cell lines (Fig. 5B). Furthermore, we designed a 3′-terminal-biotinylated-miR-223 probe that was verified to pull down circβ-catenin in NOZ or SGC-996 cells (Fig. 5C). Quantitative real-time PCR analysis of the levels of miR-223 after downregulation or upregulation of circβ-catenin. The results showed that reduced expression of circβ-catenin enhanced the level of miR-223 in NOZ and SGC-996 cells. However, overexpression of circβ-catenin decreased the level of miR-223 in GBC-SD cells (Fig. 5D, E). Taken together, the above evidence showed that miR-223 may be a direct target of circβ-catenin in GBC cells.

### Circβ-catenin regulates STMN1 expression by interacting with miR-223 in GBC cells

**Fig. 6:**
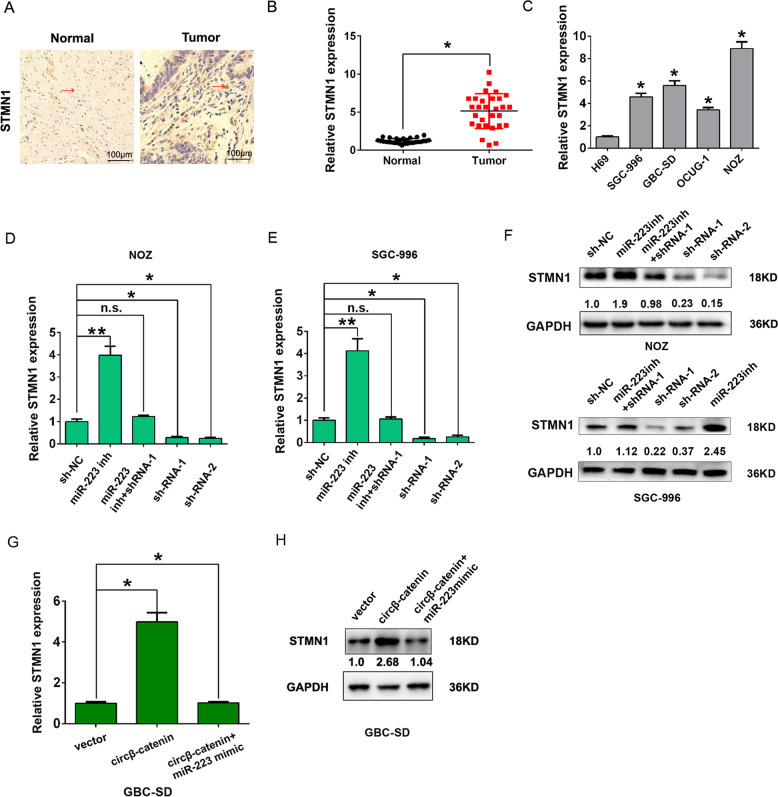
The circβ-catenin promotes STMN1 by sponging to miR-223 in GBC cells. **A** Immunohistochemical staining was shown for STMN1 expression in GBC tissues compared with adjacent normal tissues. **B** The mRNA expression of STMN1 was shown by qRT-PCR in GBC tissues compared with adjacent normal tissues. **C** The mRNA expression of STMN1 was shown in GBC cells (GBC-SD, SGC-996, NOZ, and OCUG-1) and H69 cell determined by qRT-PCR analysis. **D**, **E** The mRNA expression of STMN1 was shown by qRT-PCR in NOZ and SGC-996 cells after cells were transfected with shRNA-NC, shRNA-1, shRNA-2, miR-223 inhibitor, or miR-223 inhibitor + shRNA-1. **F** The protein expression of STMN1 was shown by western blot in NOZ and SGC-996 cells after cells were transfected with shRNA-NC, shRNA-1, shRNA-2, miR-223 inhibitor, or miR-223 inhibitor + shRNA-1. **G**, **H** The mRNA and protein expression of STMN1 was shown by western blot in GBC-SD cells after cells were transfected with pLCDH-vector, pLCDH-circβ-catenin, or pLCDH-circβ-catenin + miR-223 mimic. *P < 0.05. **P < 0.01. n.s., not significant.

Bioinformatics prediction revealed that STMN1 might act as the target effector protein for circβ-catenin/miR-223 axis (Fig. 5F). We mutated the binding sites with miR-223 in STMN1. Our results showed that the luciferase activity was reduced in STMN1-wild-type (WT) by transfecting with miR-223 mimic, but was increased by transfecting with circβ-catenin vector in NOZ and SGC-996 cells. There was no significant change in the luciferase activity of STMN1-mutant type (MUT) group in NOZ and SGC-996 cells (Fig. 5G, H). We also detected the protein and mRNA expression of STMN1 in GBC tissues by immunohistochemical (IHC) analysis and qRT-PCR analysis. The results found that the expression of STMN1 was significantly upregulated in GBC tissues compared to adjacent normal tissues (Fig. 6A, B). In addition, the expression of STMN1 was also significantly upregulated in GBC cells compared to H69 cells (Fig. 6C).

QRT-PCR analysis revealed that STMN1 mRNA expression was significantly decreased by transfecting with sh-circβ-catenin in NOZ or SGC-996 cells, but was rescued by transfecting with sh-circβ-catenin and miR-223 inhibitor (Fig. 6D, E). Western blot revealed that downregulation of circβ-catenin reduced the protein expression of STMN1, but was rescued by transfecting with sh-circβ-catenin and miR-223 inhibitor in NOZ or SGC-996 cells (Fig. 6F). Moreover, circβ-catenin overexpression transfection enhanced the STMN1 mRNA and protein, but the miR-223 mimics co-transfection rescued the mRNA and protein in GBC-SD cell (Fig. 6G, H).

### Knockdown of STMN1 inhibits cell proliferation, cell cycle progression, and Warburg effect in GBC cells

**Fig. 7:**
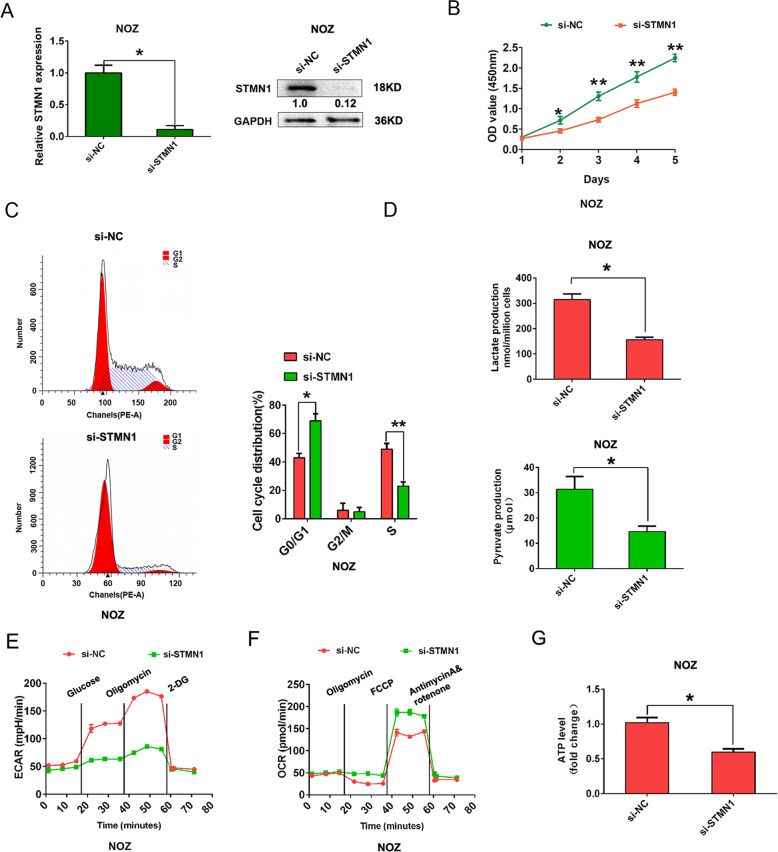
STMN1 affects cell proliferation and cell glycolysis in GBC cells. **A** The mRNA or protein expression of STMN1 was shown after NOZ cell was transfected with si-NC or si-STMN1. **B** The cell viability was detected by CCK8 assays in NOZ cells after cells were transfected with si-NC or si-STMN1. **C** Flow cytometry analysis was shown as the percentage cell phase distribution including G0/G1, S, and G2/M phases after transfection of NOZ cells with si-NC or si-STMN1. **D** The lactate production or pyruvate production levels were analyzed after transfection of NOZ cells with si-NC or si-STMN1. **E**, **F** The ECAR or OCR analysis for mitochondrial respiratory capacity was conducted using a Seahorse XFp assay after transfection of NOZ cells with si-NC or si-STMN1. **G** ATP quantity analysis revealed the ATP quantity after transfection of NOZ cells with si-NC or si-STMN1. *P < 0.05. **P < 0.01.

In addition, to investigate the functional effects of STMN1 in GBC, we knockdown STMN1 expression in NOZ cell by transfected with si-STMN1 oligos (Fig. 7A). The CCK8 assay and cell cycle analysis demonstrated that STMN1 silencing inhibited the cell proliferation ability and cell cycle progression in NOZ cells (Fig. 7B, C). Moreover, we also demonstrated that STMN1 knockdown reduced lactate production, pyruvate production, ECAR, and ATP level, while increasing OCR compared to the control group in NOZ cells (Fig. 7D–G). Thus, these findings showed that circβ-catenin/miR-223/STMN1 axis promoted GBC growth and Warburg effect in GBC progression.

## Discussion

Although the regulated mechanisms of circular RNAs’ function are not fully clear, recent studies have suggested the biological functions of circRNAs and their important regulatory roles in cancer. Some of circRNAs are identified as potential molecular markers for disease diagnosis and prognosis in human tumors [15, 16]. Circβ-catenin is first reported in human liver cancer and predominantly localized in the cytoplasm and displays resistance to RNase-R treatment. The study demonstrated that translation of the circular RNA circβ-catenin promotes liver cancer cell growth through activation of the Wnt pathway [13]. In the study, our results first showed that circβ-catenin expression was upregulated in GBC cells and higher circβ-catenin expression was significantly associated with advanced clinical stage. In addition, Kaplan–Meier survival curve and log-rank test showed that GBC patients with higher expression of circβ-catenin have significantly shorter overall survival. Multivariate Cox survival analysis showed that high circβ-catenin expression was independent prognostic factors for poor survival of GBC patients. Thus, these results first indicated that circβ-catenin expression could act as a clinical biomarker of GBC prognosis.

Further functional experiments showed that circβ-catenin knockdown inhibited cell proliferation and cell cycle progression and circβ-catenin overexpression had opposite effects. In vivo, we also demonstrated that circβ-catenin knockdown suppressed tumor growth. These evidences demonstrated circβ-catenin could affect GBC tumor growth, which suggested that circβ-catenin inhibition may be a therapeutic target of GBC. Glycolysis has been increasingly revealed as a hallmark for tumor progression in diversities of cancers [17]. CircRNAs have been identified as important regulators in tumor glycolysis and progression. Studies investigated the regulatory mechanism circ-ENO1 on its host gene ENO1 and its function in glycolysis and tumor progression. The results demonstrated that circ-ENO1 acted as a ceRNA to interact with miR-22-3p and upregulate ENO1 expression, which promoted glycolysis and tumor progression in lung adenocarcinoma [18]. Another study showed that circular RNA MAT2B was reported to promote glycolysis and malignancy of hepatocellular carcinoma through the miR-338-3p/PKM2 axis under hypoxic stress [19]. In breast cancer progression, knockdown of circDENND4C inhibited glycolysis, migration, and invasion by upregulating miR-200b/c under hypoxia [20]. In gallbladder cancer, our previous study showed that circular RNA FOXP1 promotes tumor progression and Warburg effect in gallbladder cancer by regulating PKLR expression [21]. Our results demonstrated that circβ-catenin downregulation resulted in the inhibition of the ECAR, the production of lactate and ATP, but the promotion on the OCR in GBC cells. These effects indicated that circβ-catenin promoted cell Warburg effect in gallbladder cancer.

Studies have showed that circRNAs can modulate gene expression through functioning as ceRNA including gallbladder cancer. Such as, circular RNA HIPK3 was high expression and promoted gallbladder cancer cell growth by sponging microRNA-124 [22]. Our previous study demonstrated that circFOXP1 acted as the sponge of miR-370 to regulate PKLR, resulting in promoting Warburg effect in GBC progression [21]. In the study, we found that miR-223 could form complementary base pairing with circβ-catenin. The luciferase activity was dramatically reduced in circβ-catenin-WT group compared to the control group, while the luciferase activity remained unaffected in circβ-catenin-MUT in GBC, which indicated that circβ-catenin interacted with miR-233.

STMN1 is an oncogene that is highly upregulated in many tumors and associated with tumor growth. A study found STMN1 expression acted as glycolysis-related gene signature and predicted prognosis of patients with esophageal adenocarcinoma [23]. Another study identified five glycolysis-related mRNAs (GYS2, STMN1, PPFIA4, KDELR3, and ABCB6) which were associated with patients experience biochemical recurrence (BCR) after radical prostatectomy (RP) in patients with prostate adenocarcinoma [24]. In gallbladder cancer progression, miR-223 targeted STMN1 mRNA and regulated its expression and inhibited cell growth [25]. In addition, downregulation of STMN1 in human gallbladder carcinoma was reported to inhibit tumor growth by regulating the activity of p38 MAPK kinase and p53/p21 signal pathway [26]. These above studies indicate that STMN1 functions as glycolysis-related gene, which may play important role in GBC progression. In our present study, the luciferase activity was reduced in STMN1-wild-type (WT) by transfecting with miR-223 mimic, but was increased by transfecting with circβ-catenin vector in GBC cells. There was no significant change in the luciferase activity of STMN1-mutant type (MUT) group. Moreover, circβ-catenin overexpression could enhance the STMN1 mRNA and protein expression in GBC cells by interacting with miR-223. These results indicated that circβ-catenin regulated STMN1 expression by interacting with miR-223 in GBC. We also showed that STMN1 silencing impaired the production of lactate production, pyruvate production, ATP, and ECAP, but increased the OCR in GBC cells, which suggested that circβ-catenin/miR-223/STMN1 axis could regulate cell growth and Warburg effect. Thus, we speculated that STMN1 may affect glycolysis-related pathways and we will explore it further.

In conclusion, our results first demonstrated that circβ-catenin was upregulated in GBC and regulated tumor growth and tumor Warburg effect through miR-223 upregulating its targeting STMN1. Thus, we provided that circβ-catenin could be a new potential biological marker and therapeutic target for GBC.

## Materials and methods

### Patient tissue samples

Thirty-six human GBC and adjacent normal tissue specimens were obtained from patients who underwent radical resection at Xinhua Hospital between February 2009 and February 2013. Ages ranged from 41 to 78 years (the mean value = 53.26 years). Each tissue sample was snap-frozen in liquid nitrogen for further analysis. All clinicopathological diagnoses were confirmed by two pathologists. All of tumor tissues received no radiotherapy or chemotherapy before surgery. This study was approved by the ethics committee of Xinhua Hospital. Written informed consents were acquired from patients.

### Cell lines culture and cell transfection

Four human GBC cell lines SGC-996, NOZ, GBC-SD, and OCUG-1 and a human intrahepatic biliary epithelial cell line H69 were used in the present study. GBC-SD, SGC-996, and OCUG-1 cell lines were obtained from Cell Bank of the Chinese Academy of Science (Shanghai, China). The NOZ cell line was purchased from the Health Science Research Resources Bank (Osaka, Japan). All of the cell lines were cultured in Dulbecco’s modified Eagle’s medium (Gibco, Carlsbad, CA, USA) contained 10% fetal bovine serum (Gibco, Carlsbad, CA, USA). Cells were cultured in a humidified incubator at an atmosphere with 5% CO_2_.

Two shRNAs against circβ-catenin were designed and bought from RiboBio Co., Ltd. (Guangzhou, China). Two shRNAs (shRNA-1 or shRNA-2) or shRNA-NC (sh-NC) were transfected into GBC cells using Lipofectamine 3000 (Invitrogen) following the manufacturer’s instructions. The pLCDH-circβ-catenin (circβ-catenin) was synthesized using full-length circβ-catenin and subcloned into a pLCDH-vector (vector) with the cloning sites BamHI/EcoRI (GENESEED, Guangzhou, China). MiR-223 mimic (5′-UGUCAGUUUGUCAAAUACCCCA-3′), miR-223 inhibitor (5′-UGGGGUAUUUGACAAACUGACA-3′), and miRNA control (5′-CAGUACUUUUGUGUAGUACAA-3′) were designed and bought from RiboBio Co., Ltd. (Guangzhou, China). Transfection of STMN1 siRNA (5′-AAGAGAAACUGACCCACAA-dTdT-3′) and scramble control (5′-UUCUCCGAACGUGUCCGU-dTdT-3′) (RiboBio Co., Ltd., Guangzhou, China) were performed using Lipofectamine 2000 Transfection Reagent (Invitrogen).

### RNA extraction and quantitative real-time PCR (qRT-PCR)

Total RNAs were extracted from GBC tissues and cell lines using TRIzol reagent (Life Technologies, Carlsbad, CA, USA) according to the manufacturer’s instructions. The cDNAs were synthesized from total RNA using Prime-Script RT reagent kit (TaKaRa, Japan). U6 and glyceraldehyde 3-phosphate dehydrogenase (GAPDH) were utilized as controls. All primers were designed and purchased from Sangon Biotech (Shanghai, China). The primer for circβ-catenin forward: 5′-AGTGCTGAAGGTGCTATCTGT-3′, the primer for circβ-catenin reverse: 5′-AGGTAAGACTGTTGCTGCCA-3′; the primer for STMN1 forward: 5′-GCCTGTCGCTTGTCTTCT-3′, the primer for STMN1 reverse: 5′-TCATGGGACTTGCGTCTT-3′; the primer for GAPDH forward: 5′-CAACAGCCTCAAGATCATCAGC-3′, the primer for GAPDH reverse: 5′-TTCTAGACGGCAGGTCAGGTC-3′; the primer for U6 forward: 5′-CTCGCTTCGGCAGCACA-3′, the primer for U6 reverse: 5′-AACGCTTCACGAATTTGCGT-3′; the 2^−ΔΔCt^ methods were used to analyze the quantitate mRNA expression.

### Nuclear-cytoplasmic fractionation

Cytoplasmic and nuclear RNAs were isolated using NE-PER Nuclear and Cytoplasmic Extraction Reagents (Thermo Scientific, USA) following all manufacturer protocols. We followed this experiment with qRT-PCR analysis.

### RNase R treatment assay

GBC cells were collected and divided into groups, respectively. Cells were treated with or without RNase R (3 U/μg; Epicentre, Madison, WI, USA) at 37 °C for 20 min. Then, RNA was purified with phenol, chloroform, and isoamyl alcohol. Circβ-catenin stability was determined by detecting circβ-catenin expression by qRT-PCR.

### Fluorescence in situ hybridization (FISH)

Cells were fixed in 4% paraformaldehyde and washed with PBS for 30 min. After treating with FISH probes specific for biotin-labeled RNA probe (circβ-catenin: ACT + TCAAA + TACCCAGCT + TC + TACAAT) (RiboBio Co., Ltd., Guangzhou, China) in hybridization buffer overnight, cells were washed with PBS three times and then blocked with HRP blocker. Cells were hybridized in hybridization buffer and 4 ng/µl of RNA probe in 2 × SSC plus 50% formamide 60 °C overnight. Finally, signals were detected using a tyramide-conjugated Alexa 488 fluorochrome TSA kit (Life Technologies).

### Cell viability assay

Cell viability was measured using cell counting kit-8 (CCK8, Dojindo Laboratories, Japan) following the manufacturer’s instructions. In brief, cells (2 × 10^3^) were seeded into 96-well plates before transfection. After transfection at 1, 2, 3, 4, and 5 days after cell transfection, a CCK8 solution (10 μl) was added to each well. Then, after 2 h of incubation at 37 °C, absorbance at 450 nm was determined using Spectra Max 250 spectrophotometer (Molecular Devices, Carlsbad, CA, USA).

### Flow cytometry analysis

Flow cytometry was used to analyze the cell cycle of GBC cells. After cell transfection at 48 h, GBC cells were immobilized using 80% ice-cold ethanol. RNase (Sigma) was used to remove RNA at a concentration of 2 mg/mL for 30 min at 37 °C. 20 mg/mL propidium iodide (PI; Sigma) was added to mark the DNA content for 20 min at 37 °C. The percentages of GBC cells in different phases of the cell cycle were analyzed by the flow cytometer.

### Western blotting analysis

Cells were harvested and lysed using lysis buffer (Beyotime, Shanghai, China). The proteins were separated by 8–10% SDS-PAGE and transferred to polyvinylidene difluoride (PVDF) membranes (Merck Millipore, Germany), which were blocked in 5% nonfat milk and then incubated with primary antibodies against STMN1 (1:2000, Abcam, USA), β-catenin (1:1000, Protein Tech, 51067-2-AP, China), or GAPDH (1:1000, Protein Tech, 10494-1-AP, China) at 4 °C overnight. Then, the horseradish peroxidase-conjectured goat anti-rabbit secondary antibody (1:1000, Abcam, UK) was incubated with the membranes. Finally, the protein bands were visualized by using an electrochemiluminescence (ECL) system and the gray values were measured by Image J software.

### Biotin-coupled probe pull-down assay

The biotin-labeled miR-223 probe (UGUCAGUUUGUCAAAUACCCCA) and the negative control probe were designed and synthesized by Gene Pharma Biotech (Shanghai, China). Briefly, NOZ and SGC-996 cells were fixed, lysed, and centrifuged. Then, Streptavidin-coupled Dynabeads (Invitrogen) were washed and resuspended in the buffer. Then, an equal volume of the biotin-labeled miRNAs was added in the buffer. After incubating at room temperature for 10 min, the coated beads were separated with a magnet for 2 min and washed three times. The isolated RNAs were then subjected to qRT-PCR analysis.

### Bioinformatics analysis

circβ-catenin sequence data were obtained from circBase (http://www.circbase.org/). The target miRNAs of circβ-catenin were predicted with circular RNA interactome (https://circinteractome.nia.nih.gov).

### Luciferase reporter assays

The sequence of full-length circβ-catenin 3′UTR with WT or MUT binding sites for miR-223 was inserted into psicheck2 vector (Promega, Madison, WI, USA). The circβ-catenin-WT/MUT plasmids were co-transfected with miR-NC or miR-223 mimic into NOZ and SGC-996 cells. STMN1 3′UTR or corresponding MUT were designed and was inserted into psicheck2 vector (Promega, Madison, WI, USA). The STMN1-WT/MUT plasmids were co-transfected with vector, circβ-catenin, miR-223 mimic or circβ-catenin + miR-223 mimic in NOZ or SGC-996 cells. The luciferase activity normalized to Renilla luciferase activity was analyzed via a Dual-Luciferase reporter assay system (Promega, Madison, WI, USA).

### Tumor xenograft experiments

BALB/c nude mice (3 weeks old) were purchased from Vital River Laboratory Animal Technology (China). NOZ cells (1 × 10^6^) stably expressing circβ-catenin shRNA or control group were subcutaneously injected into either side of the flank area of 3-week-old BALB/c athymic nude mice. Tumor size or volume was measured every week. All mice were euthanized after 4 weeks and subcutaneous tumors were dissected and collected. Tumor volume (*V*) was measured weekly and calculated using the equation *V* = (*a* × *b*^2^)/2, Final tumor weights were measured and tumor samples were subjected to IHC staining. All animal experiments were performed in the animal laboratory center at Xinhua Hospital and conformed to the Guide for the Care and Use of Laboratory Animals published by the US National Institutes of Health (NIH publication number 85-23, revised 1996).

### Immunohistochemistry (IHC)

Tumor tissues were fixed using 4% paraformaldehyde and embedded in paraffin. The samples were then incubated with primary antibody against Ki-67 (1:600, Abcam, USA), treated with secondary antibody for 30 min, and stained with diaminobenzidine (DAB) until brown granules appeared.

### Extracellular acidification rate/oxygen consumption rate measurements

The extracellular acidification rate (ECAR) or oxygen consumption rate (OCR) was measured using a Seahorse XF24 analyzer (Seahorse Biosciences). Briefly, after cell transfection, 1 × 10^4^ cells/well was cultured in a 96-well XF microplate (Seahorse Biosciences) overnight. Cells were washed before incubation with assay medium (0.5 mL) for 1 h at 37 °C. Then, cartridge ports A, B, and C were loaded with 75 μL of glucose (80 mM), oligomycin (9 μM), and 2-DG (1 M), respectively. The ECAR after glucose treatment indicated the glycolysis rate. The ECAR after oligomycin treatment indicated the glycolysis capacity.

### Statistical analysis

The data was analyzed by SPSS 20.0 (IBM Corporation, Armonk, NY, USA) and GraphPad Prism 6.0 (GraphPad Software, La Jolla, CA, USA) and are shown the mean ± standard deviation (SD). The differences between the two unpaired groups were assessed using unpaired Student’s *t*-test. Differences between multiple cell lines were assessed using one-way ANOVA followed by the least significant difference (LSD) test. Correlations between circβ-catenin expression level and clinicopathological characteristics were analyzed by Pearson’s correlation test. Multivariate Cox proportional hazards regression models were used to identify factors associated with survival. The survival analysis was conducted with the Kaplan–Meier plots and the log-rank test. *P* values < 0.05 were considered as statistical significance.

## Data Availability

The datasets used and/or analyzed during the current study are available from the corresponding author on reasonable request.
